# Exploring Agency, Autonomy and Authority Among Lead Tissue Viability Nurses: A Qualitative Study

**DOI:** 10.1002/nop2.70401

**Published:** 2025-12-21

**Authors:** Fania Pagnamenta, Tim Rapley, Monique Lhussier

**Affiliations:** ^1^ The Newcastle Upon Tyne Hospitals NHS Foundation Trust Newcastle upon Tyne UK; ^2^ Northumbria University Newcastle upon Tyne UK

**Keywords:** authority, autonomy, Lead Tissue Viability Nurses, nurse, power, professional identity

## Abstract

**Aim:**

To understand Lead Tissue Viability Nurses' agency, autonomy and authority to perform their role.

**Background:**

Lead Tissue Viability Nurses need both clinical autonomy and organisational authority to improve wound care, patient safety, and cost‐effectiveness. Their role goes beyond bedside care to include strategic decisions shaping policy, resources, and quality initiatives. Despite this, the role is often misunderstood and under‐recognised. This study, based on the four pillars of advanced nursing practice—clinical practice, education, research, and leadership—examines the challenges they face.

**Design:**

A qualitative study.

**Methods:**

Interviews with six Lead Tissue Viability Nurses working in large tertiary care organisations. A thematic analysis was undertaken.

**Results:**

The results aligned with the four pillars of advanced nursing practice. Six sub‐themes emerged, reflecting the skills, knowledge, and influences involved, and critically, the challenges faced in meeting role expectations: Clinical Practice—working with clinical specialties (a); Education—supporting generalist nurses (b); Leadership—collaborating with dressing companies (c) and national bodies (d); Research—partnering with clinicians (e) to implement national guidance (f).

**Conclusion:**

Our study highlights the restricted agency that constrains their capacity to meet the complex demands of the role. Their specialised knowledge frequently remains unacknowledged and underutilised, while their contributions are often marginalised within organisational discourse. To attain the level of recognition commensurate with this senior nursing position, their distinct professional and organisational identity should be clearly articulated.

**Implications for the Profession and/or Patient Care:**

Lead Tissue Viability Nurses should be acknowledged for their advanced practice role, reflecting the specialist expertise and leadership they bring to patient care. Additionally, they must be granted the agency, autonomy and authority necessary to implement best practice.

**Impact:**

Ensuring that Lead Tissue Viability Nurses can execute their duties effectively and achieve high‐quality outcomes will directly influence patient care.

**Patient or Public Contribution:**

No patient or public contribution at this stage, as this study was about exploring the nurses' organisational role from their perspective.

## 1 Introduction

Lead Tissue Viability Nurses (LTVNs) are registered nurses who specialise in wound care and manage teams of Tissue Viability Nurses (TVNs) at different stages of education and training; as a team, they focus on preventing damage to the skin and underlying tissues and facilitate healing in wounds where a complication has prevented the normal healing process (Ousey et al. 2014). The role is nurse‐led and spans across all medical disciplines, from the cradle to the grave. LTVNs and their teams collaborate with medical colleagues to care for complex wounds but generally work without direct medical supervision. Patients benefit greatly from the expertise of tissue viability nurses, who help prevent and manage complex wounds (Welsh 2017).

In the majority of cases, their line managers are senior operational nurses with limited wound care knowledge. LTVNs advise their organisation on the selection and procurement of a range of medical devices, such as dressings, therapy mattresses and beds, booties, and other products as well as setting clinical standards, competencies, policies, and guidance for their multi‐professional colleagues. In the UK, LTVN is an advanced role as defined in the Standards for Advanced Nursing Practice (RCN 2023) and is embedded in its four pillars: clinical practice; education; leadership and research.

They require agency, autonomy and authority to undertake their role. Agency, autonomy, and authority represent distinct yet interrelated concepts within professional nursing practice. Agency refers to the capacity of nurses to act intentionally and make choices within the structural and cultural constraints of healthcare systems (Lopéz‐Deflory et al. 2023). Autonomy builds on this by emphasising the freedom to exercise such agency independently, allowing nurses to make discretionary and binding decisions consistent with their scope of practice (Mrayyan et al. 2024). In contrast, authority denotes the formally sanctioned power to influence decisions, allocate resources, and direct others, as recognised in professional codes and leadership standards (ANA 2025).

Internationally, the role of the LTVN shares a common foundation in its core purpose and clinical expertise, primarily centred on complex wound management and prevention. Despite these similarities, variations exist in professional autonomy, educational pathways, and integration within healthcare systems.

Core responsibilities include assessing and treating complex, non‐healing wounds such as pressure ulcers, leg ulcers, and surgical wounds. LTVNs require advanced skills in wound and risk assessment, appropriate dressing selection, and the use of pressure‐relieving equipment. A universal aspect of the role is educating generalist nurses and other healthcare professionals to enhance knowledge and practice in wound prevention and management.

Key differences arise in clinical autonomy and educational requirement. In some healthcare systems, LTVNs exercise considerable independence in product selection and care planning, whereas in others, physician authorisation is mandatory for specific interventions, such as prescribing treatments or performing debridement. Educational pathways also vary significantly; for example, whilst the UK lacks a standardised national qualification in tissue viability (Munro and Beck 2021), other countries offer structured postgraduate programs, such as those provided by the Finnish Wound Care Society or the wound, ostomy, and continence certification in the United States.

Working at advanced nursing practice level (RCN 2023), LTVNs are expected to ‘exercise autonomy and decision making in a context of complexity, uncertainty and varying risk, holding accountability for decisions made’ (HEE 2017, 8). In order to work at this advanced level, LTVNs are to be educated at Masters level; to have specialist knowledge and skills to prevent and manage wounds, and to identify, appraise, analyse, and implement evidence findings into clinical practice across all specialities under their remit. They are skilled in a wide range of clinical skills, including conservative sharp debridement, a surgical procedure to remove necrotic tissue from a wound bed (Harris et al. 2018). They are also required to have business acumen as procurement is one of the essential aspects of the role (Ousey et al. 2014).

In 2006 Austin et al. published two seminal papers on the relationships that TVNs have with other health‐care professionals and its impact on the role in the UK (Austin et al. 2006). They explained that TVNs are recognised as having expertise in a given field, which they use to develop the practice of others, and they share many of the characteristics of entrepreneurs, which they use to develop services related to their speciality (Austin et al. 2006). Their study found that TVNs were the main drivers for service development, however they encountered many difficulties when introducing new ideas, namely they lacked agency, authority and autonomy to bring about change and were dependent on ‘cultivating relationships’ with more powerful health professionals or manager to achieve their vision. Building on the work of Austin et al. (2006) and more recently, Jakobsen et al. (2021), this paper reports on the findings of a study that explored if, fifteen years later, LTVN have been able to develop the necessary authority and autonomy to fulfil their advanced nursing role.

## 2 Background

Wound care is developing to a speciality in its own right. Wound care was one of the first tasks for nurses, particularly when caring for casualties of war and remained as such for decades, mostly under doctors' orders (Pijl‐Zieber 2013). Dressing materials were simple and not always efficient; the outcomes improved dramatically with the post‐war use of antibiotics; at that time, it was thought that a wound would heal well if it were allowed to dry out and form a scab. Winter's seminal work on pigs, discovered that wounds healed best if kept moist and this finding revolutionised clinicians' understanding of wound healing. As the understanding of the science behind wound healing has continued to develop, researchers have applied some of their findings to the development of new dressings. Dressing materials have become technologically advanced, selecting a dressing is now a complex and costly affair. Wound care is no longer seen as a delegated task but a key responsibility and principal element of care for nurses (Welsh 2017) for which they are held accountable (NMC 2018). The support for the nurses who undertake day‐to‐day wound care comes from TVNs rather than medical staff. TVNs deliver formal and ad hoc training and education alongside their busy clinical practice schedule.

The Department of International Trade (2018) suggests that there are estimated 748 TVNs in the UK, these TVNs work in small teams, led by a LTVN. With an estimated 3.8 million patients living with a wound (Guest et al. 2020), each TVN supports nursing staff with about 5000 patients at any given time. Tissue Viability is an under‐resourced speciality (Ousey et al. 2014) with low profile, possibly because it is nurse‐led without medical input and because of a poor understanding of the width and depths of the role. Austin et al. (2006) observed how LTVNs have to cultivate relationships with more powerful allies to get what they want because the role itself does not give them the level of autonomy and authority required to practice most effectively. The work undertaken to gain the agency, authority and autonomy necessary for their role was also observed in another UK study of how LTVNs selected dressings for Wound Management Formularies (WMFs) (Pagnamenta et al. 2025—in review). A follow‐up study was conducted in 2022 to explore this issue further, to explore, whether LTVNs have the necessary authority and autonomy to fulfil their advanced nursing role.

## 3 The Study

### 3.1 Aim and Research Question

The aim of the study was to understand what agency Lead Tissue Viability Nurses' have to perform their role. The research question was: ‘What agency do Lead Tissue Viability Nurse have to perform their role effectively?’

The objectives were to examine the challenges LTVNS face in exercising clinical authority and organisational influence across the four pillars of advanced nursing practice (clinical practice, education, leadership and research); to identify the skills, knowledge, and influences required for LTVNs to meet role expectations; to investigate organisational and professional factors that constrain LTVNs' autonomy and recognition within healthcare systems.

## 4 Methodology

### 4.1 Design

The study is a qualitative design based on semi‐structured interviews. This study was informed by a constructivist approach, which recognises that people construct their knowledge based on their own experiences and that each person may experience reality in a different way. The selection of a qualitative methodology facilitated the exploration of complex experiences, attitudes, and motivations from the participants that eluded quantitative representation (Moorley and Cathala 2019). The study is reported using the Standards for Reporting Qualitative Research (SRQR) framework (see Appendix 1).

### 4.2 Theoretical Framework

This study is underpinned by a constructivist paradigm, which posits that knowledge and meaning are actively constructed through individual experiences rather than discovered as objective truths. Constructivism assumes that reality is subjective and multiple, shaped by social interactions and personal interpretations (Williamson 2018). This approach was particularly suited to exploring the complex professional experiences of LTVNs, as it allows for an in‐depth understanding of how they perceive and enact their roles within diverse healthcare contexts.

### 4.3 Sampling and Recruitment

Purposive sampling was employed, a non‐probability technique in which participants are deliberately selected based on predefined criteria aligned with the study's objectives. This method is particularly useful for obtaining in‐depth insights from a targeted group, ensuring that those included possess characteristics or expertise directly relevant to addressing the research question (Shorten and Moorley 2014). Purposive sampling was chosen because the study aimed to capture perspectives from LTVNs with specific roles and experiences within large NHS Trusts, many of which span both acute and community care. Such participants were considered most likely to provide rich, contextually grounded information that could not be obtained through random sampling. Participants were invited via email by the first author, who is herself an LTVN, reinforcing the credibility of recruiting individuals best suited to address the research question (Moorley and Cathala 2019).

### 4.4 Sample Size and Power

Six LTVNs were recruited from ten major NHS teaching and research trusts in England, which together account for about 10% of the NHS budget and employ 11% of its workforce. Of the nine LTVNs in these trusts, six consented to participate. This sample is considered adequate for qualitative research, as saturation in homogeneous groups often occurs with fewer than ten participants (Moser and Korstjens 2018). Given the study's exploratory nature and the small, well‐defined population, six participants were sufficient to ensure credible findings (Moorley and Cathala 2019). The participants were familiar to the lead author (herself an LTVN), as this group of senior nurses is a small, well‐defined community within the UK.

### 4.5 Population and Sample

All participants were Lead Tissue Viability Nurses, with over 8 years' experience in their role. They were all educated at Masters to PhD level. They received a written participant information sheet, were offered 1 week to decide if they wished to participate and signed a written consent form. To preserve their identities, all the interviewees are represented as female and were allocated a number (P1 to P6).

### 4.6 Data Abstraction

The transcripts were uploaded onto Nvivo, a qualitative data analysis software, which allows for cross‐case thematic analysis. Transcripts were reviewed for accuracy against the audio files. Key information relevant to the research objectives was systematically abstracted, including participant demographics, contextual details, and thematic content. All identifying information was removed prior to coding and interpretation.

### 4.7 Inclusion/Exclusion Criteria

*Inclusion Criteria*: LTVNs who lead their services within a large tertiary NHS Trust in England.

*Exclusion Criteria*: All other TVNs who do not hold a service leadership role; LTVNs working in non‐tertiary Trusts or outside England.

### 4.8 Data Sources/Collection

Interviews were conducted virtually using Microsoft Teams, providing a flexible and accessible platform for participants. The discussions explored several key areas of professional practice. First, they examined the participants' clinical expertise and how this informs day‐to‐day decision‐making and patient care. Second, the interviews delved into their ongoing professional development, as well as their role in supporting the growth of generalist nurses within the team. Leadership and collaborative practice were also central themes, focusing on how participants foster teamwork, influence change, and build interprofessional relationships. Finally, the conversations addressed their capacity to drive quality improvement initiatives and contribute to the development of evidence‐based practice, highlighting strategies for enhancing patient outcomes and service delivery.

The interview schedule was developed to provide consistency and structure across all research interviews. It outlined the key topics, questions, and prompts aligned with the study objectives, while allowing participants the flexibility to elaborate on their experiences. This approach ensured comparability of responses and maintained focus on the research aims. A pilot was not conducted due to time and resource constraints, as well as the need to adhere to the project timeline. Limited participant availability further reduced the feasibility of a pilot phase.

#### 4.8.1 Data Management and Confidentiality

Access to the data was restricted to the research team only, ensuring that no unauthorised individuals could view or handle the information. All data was stored securely on encrypted, password‐protected systems within the organisation's network, complying with institutional and GDPR standards. During interviews, confidentiality was maintained by conducting sessions in private settings, anonymizing participant identifiers, and ensuring that recordings and transcripts were stored in secure folders. Privacy was safeguarded throughout the process by using coded identifiers instead of personal details and limiting access strictly to the research team involved in analysis.

### 4.9 Data Analysis

The data underwent thematic analysis following Braun and Clarke's six‐phase framework (2006). This process included: familiarising with the data and noting initial ideas; generating codes across all transcripts; identifying potential themes by grouping similar codes and collating relevant data; reviewing themes against the dataset; refining and exploring relationships between themes; and finally, defining themes and conducting detailed analysis. The approach emphasises reflexivity and rigour, both critical for ensuring credibility. Later iterations by Braun and Clarke (2019, 2021) introduced refinements, such as distinguishing reflexive thematic analysis from coding reliability approaches, which can add complexity to the process. The lead author undertook the data collection and analysis, supported by the research team. Six core themes emerged and are described in the findings below, aligned to the four pillars of practice advanced nursing practice (RCN 2023).

### 4.10 Ethical Considerations

Northumbria University Ethics Committee (Submission Reference 22867) granted ethical approval.

#### 4.10.1 Rigour

The research team have substantial expertise in qualitative inquiry, underpinned by a commitment to methodological rigour. Their experience encompasses the development of robust qualitative frameworks, the facilitation of in‐depth interviews, and the application of systematic and transparent approaches to thematic analysis.

A copy of the recording as well as the verbatim transcription was sent to each participant, giving them the opportunity to listen/read what was said and comment if they so wished. None of the participants requested amendments. Several practical strategies were used to strengthen rigour in this study beside returning interpretations to participants for validation; comprehensive records of decisions, data collection, and analysis processes were kept; bar the lead author, the research team and co‐authors are academics with no clinical background—they reviewed and challenged interpretations. The lead author maintained a reflective journal to document assumptions and potential biases. The analysis was discussed and reviewed with non‐clinical research team members, to share and exchange interpretations of key issues emerging from the data to ensure rigour and also to ensure that insider–outsider relationship between the first author, a LTVN herself and the participants were minimised as much as possible.

## 5 Findings

There are four pillars of practice, which LTVNs, as specialist nurses must have as part of their core role and function (RCN 2023). The findings have been aligned to these four concepts that detail the skills, knowledge and level of influence that is required to be an efficient LTVN.

### 5.1 Clinical Practice: Interfacing With Clinical Specialities

Tissue Viability is a nurse‐led service, which has a distinct way of working, as explained below,

We are a nurse‐led service and I promote that massively because I cannot think of any other CNS [Clinical Nurse Specialist] in the NHS that works as autonomously as TVNs. There is no other CNS that work without a doctor […], as a collective, we should shout out ‘this is a 100% nurse‐led service’. (P6)

This independent way of working is specific to TVNs as all other specialities work directly under the clinical supervision of a medical practitioner. For instance, vascular nurses work under the direct supervision of a vascular consultant, and so forth.

LTVNs are knowledgeable and experienced in wound care and work across specialities, from paediatrics to elderly care, without medical supervision. They not only provide care for patients with complex wounds but also develop protocols and guidelines that ensure consistency and effectiveness across multiple specialties, organisational units, and established norms.

As [TV] nurses, we're very proactive and we know what we want, and we know when we want it, why we want it etc. And we understand the process, but I don't think it's clear to anybody else. [P3]

However, LTVNs are mostly line‐managed by nurse managers who do not have wound care expertise and often not understand the challenges that LTVNs are faced with, and these will be explored in the sections below.

### 5.2 Education, Developing Self and Others: Interfacing With Generalist Nurses

The participants agreed that LTVNs have a sound wound care knowledge that includes ‘a strong stomach, because of the aromas’ of wound care and ‘need to have a poker face, rather than a happy smiley face all the time because not all of what we do is happy smiley’, alluding that wound care is at times mal‐odorous, painful, and uncomfortable for the patient [P1]. Most LTVNS are educated at Masters level, have knowledge of litigations and the law [P6]; they have management and leadership qualifications ‘understand QI methodologies’[P2]; ‘understand risk and governance’ [P2]; ‘understand how the NHS works’[P4]; ‘what roles people have in the NHS’ [P3]; have knowledge of supply chains, procurement, marketing, and business [P6]. LTVNs engage with research. Additionally, P1 said,

What is a Tissue Viability Nurse? We need exceptional communication skills for all disciplines, and we need to have some—I'm going to say it, I'm sorry—some balls inside you. You need to be bolshy but polite. You need to be confident. You need to be like a swan. Everything exterior is calm and smiling and charming. Inside, you are screaming ‘you need to just do it this way’. Erm, so we need to be great communicators.

In the above quote, we see that LTVNs believe they require strong interpersonal skills that demonstrate confidence at face value; indeed, making changes to clinical practice that are experiential rather than evidence based requires considerable courage, as often other professionals have their own experiences to base their practice upon. LTVNs need to have a wider angle view on the issues at hand, which will be explored further in the next sections. However, interestingly, internally, they often voice a lack of confidence and a fear that they will not be listened to and that their decisions might not be upheld by others they work with.

They also need to actively support the clinical skills and knowledge of those they work with. P3 explains the extent of wound care training and education that they are required to undertake.

You know, with four and a half thousand [nurse] registrants within my organisation, trying to keep them up to date with the policies, with the procedures, with the teaching, with the training, with everything else that goes with this… You no sooner get it sorted and something else is changing. And then something else is changing.

Centrally, LTVNs have to rely on private companies that provide the dressings to provide education and training to generalist nurses, and this has to be factored in the decision making process whether to list a product or not. It creates tension for LTVNs as not only do they have to balance dressing effectiveness with price, but also with what the company can provide with regards to an adequate training offer. Often, only big companies can provide the scale of training required for a large organisation to ensure suitable coverage.

### 5.3 Leadership and Collaborative Practice: Interfacing With Dressing Companies

LTVNs are responsible for the compilation of a Wound Management Formulary (WMF) that is to select a limited range of dressings from a vast array available on the market, to be used within their organisation. They have to monitor adherence to the WMF and ensure that compliance is maintained. The market is lucrative and very competitive, and companies employ representatives to sell dressing products to the NHS.

The participants explained that they have to ‘gate‐keep’ [P4], preventing companies from advertising and selling their non‐WMF products directly to clinicians. This causes challenges for TVNs, as P2 explains below,

Sometimes they [private companies] use different routes; they go above the Head of Nursing; they go to the Chief Nurse and say ‘Tissue Viability is not using our recommended dressings; these are cost saving…’ Sometimes they go through procurement but luckily, we have got a protected formulary; they [procurement] cannot change the formulary without my permission.

As such, LTVNs are subjected to significant pressures. Private company sales representatives bypass WMF gatekeepers and question the LTVNs decisions with their line manager. The fact that the Chief Nurse in this example agrees to meet company representatives is an indication that TVNs do not often have the necessary autonomy, credibility and trust that is required to undertake their core responsibilities. Given their remit and competing demands on time, decision makers that companies try to reach to overrule LTVNs, such as a Chief Nurse, may not understand that the decisions for listing a product versus another are multi‐faceted and requires detailed explanation that goes further than cost considerations exercise. As seen above, the portfolio for training generalist nurses is beyond the capacity of small TV teams.

### 5.4 Leadership and Collaborative Practice: Interfacing With National Bodies

At a national level, LTVNs are rarely included in key decision‐making strategies, for example, in dressing procurement. In the UK, in acute care but also in some community settings, dressings are procured via NHS Supply Chain (NHSSC), an organisation that was set up by the Department of Health and Social Care but is now run by a number of private logistics organisations (Hall et al. 2020). NHS Trusts are actively encouraged to purchase their dressings via this route and as an interviewee noted ‘any dressing that you need that is not in the NHS supply chain, it's almost impossible to get’ [P2]. The main benefit of the NHS supply chain is that they deliver the required supplies directly to each ward and department.

NHSSC initiated a process of rationalisation where the determinant factor is price rather than cost‐effectiveness (Hall et al. 2020). This creates tensions for LTVNs. P6 struggles to understand ‘decisions that are made without a strong clinical rationale’ given that there extensive ‘talent and knowledge and expertise in tissue viability’. Such experience is not currently used to make decisions that are primarily driven by clinically acceptability. The impact on LTVNs has been considerable, for instance,

Impact on me as a clinician? It's been… frustrating; time consuming; disappointing. As a clinician, I feel that our main focus is our patients and that the decisions NHSSC makes are based on considering dressings as a commodity. I don't think they understand that they are used as part of therapy and that that has an impact on patients in the process and that it's very frustrating. As a nurse specialist, who's been working with a lot of these products for years, to [be imposed] the lowest decision point [i.e., unit cost]. [P5]

Given LTVNs knowledge, experience, and an extensive deep understanding of how these products work; being imposed ‘the lowest decision point’ and not being consulted or listen to is problematic for them. Tissue viability is about patients and yet, in what the supply chain approach has taken, LTVNs ‘cannot see patients’ P6 in there at all. Indeed, patients are not visible within that whole process.

### 5.5 Research, Improving Quality and Developing Practice: Interfacing With Other Clinicians

LTVNs have to tap into sources of power within their organisations to gain the autonomy and authority to act. To do so, they have to forge links with decision making allies in their organisation, such as medical staff. P1 said,

My Vascular Access Nurse is very good… Has a really good working relationship with the anaesthetist, so if we needed to change a film dressing for instance, we can do it through the anaesthetist, for them to help us with the surgeons. And that's a bit of a roundabout way to get something in, but sometimes you have to frame these channels because it's the only way you can get things to change.

The above quote demonstrate how, in order to create change, LTVNs have to seek indirect routes, in this case the anaesthetist. P3 explains that ‘we kind of go round about the houses; it's the only way, unfortunately, we can get some things changed’. Direct conversations are often not welcomed; the inconsistency of support by line managers is highlighted in the except below, where the Chief Nurse should have been aware of the challenging facets of the role and supported her decision‐making; instead P3 explains how she was ‘summoned’,

We changed one of our film dressings […] and received a number of emails, quite angry emails, almost bullying emails. ‘This is the surgeon's choice; this has nothing to do with tissue viability’ And ‘we are escalating this higher’ and it did go higher, all the way up to our Chief Nurse and I was summoned to explain myself.

Such an experience of bullying and unpleasant behaviour is shared by a number of TVNs interviewed. The lack of respect for the role and the lack of support brings about considerable emotional labour, ‘it was a very very stressful period of time’ [P3].

In order to minimise such exposure, LTVNs find ways to collaborate with other TVNs and form groups so that the responsibility for any decisions made are fronted by ‘a group’, an impersonal entity, rather than that one individual and are thus able to make the same decisions at a reduced emotional labour, as illustrated in the following quote,

I've got more authority to change things now, because I've got this ICS‐wide wound care formulary group behind me and it's all great clinicians; it's all TVNs like myself and […] I feel that we do have more authority than we ever used to. I don't say it's any easier, because you've still got red tape to go through, but it feels probably better now than it did [in the past]. [P1]

Thus, whilst there remain administrative processes to go through, seen as ‘red tape’, the benefit of having more decision‐making power without antagonism outweighs the bureaucratic steps to ‘get what one wants’ [P1].

### 5.6 Research, Improving Quality and Developing Practice: Interpreting and Implementing National Guidance

LTVNs also undertake thorough assessments of the dressings available and consult widely in their organisation and beyond to make decisions; at times, they appear to go against national guidance. For example, P4 relates that she was summoned to the Chief Nurse's office,

Have you seen this NICE recommendation for this particular dressing? Have you looked at it well? The company has sent them […] the ‘NICE guidelines’. They [Chief Nurse Office] said, ‘pressure ulcers are not reducing’, why are you not trying this?

In this context, P4 had to justify that the approach to pressure ulcer prevention she had taken continued to be the most cost‐effective and that pressure ulcers developed for a number of reasons, such as staffing skill mix, equipment, and education that were outside her control. LTVNs have to effectively advocate for specific ways of working, to continually work to demonstrate good clinical reasoning for working with and at times, deviating from, regulatory directions.

## 6 Discussion

The authority of LTVNs in decision‐making is inherently precarious, as their judgements often intersect with competing commercial and clinical interests. Companies frequently contest decisions perceived as detrimental to their business outcomes, while medical colleagues and senior nursing leadership may challenge LTVNs when external complaints arise, revealing a systemic vulnerability in their role. This dynamic forces LTVNs to pursue organisational change through indirect strategies, leveraging alliances across clinical teams and networks of TVNs, rather than through formal hierarchical channels. Such reliance on informal influence underscores the tension between professional autonomy and institutional politics. In this context, the adage ‘no one is a prophet in their own land’ aptly captures the paradox of expertise without agency, autonomy or authority, highlighting the cultural and structural barriers that constrain LTVNs' capacity to lead transformative practice within their own organisations.

Autonomy does not necessarily result in authority for decision making or freedom to act. Indeed, this study observed how on one hand, LTVNs require high levels clinical skills, and knowledge pertaining to organisational support functions, such as procurement, business, and law. LTVNs are generally educated at Masters level as per Standards for Advanced Nursing practice (RCN 2023) which reflects the participants studied, as they need skills to critically examine evidence and extract meaning; exhibit capacity for critical thinking and analysis; use knowledge to solve problems creatively; be able to deal with complex, unpredictable issues and make informed decisions based on incomplete information (Madi et al. 2019). On the other hand, in their organisations, LTVNs have limited influence over their more powerful colleagues (be they medical staff or nursing managers) and nationally, they are not listened to when key decisions are made to promote cost savings, for example in the procurement of medical devices.

This situation points towards a dichotomy between what LTVNs require to undertake the job and what they have the freedom to act on. For instance, LTVNs have to develop a WMF for their organisation, limiting the range of dressings that are used. The benefits for having a reduced list of dressings are considerable, from negotiating powers over price to give the opportunity to junior staff to know a few products well, rather than be overwhelmed by choice. Arguably, LTVNs make decisions based on incomplete information as evidence for the use of any dressings remains scant (Pagnamenta 2017; Welsh 2017) and in their suitability appraisals, they have to include aspects such as procurement routes, costs, training and support offered by the company (Pagnamenta et al. 2025—in review). Clinicians with more power have their ‘likes and dislikes’; their dressing selections equally lack foundation in research evidence and are often based on ritualistic practice (Welsh 2017) and they do not always understand the need to select dressings that fit a wide range of clinical situations.

The literature positions power as a prerequisite for effectiveness (Kanter 2017; Manojlovich 2007), yet LTVNs operate in a paradoxical space, expected to lead change while lacking formal authority. This contradiction is not benign; it forces LTVNs to rely on informal strategies such as ‘working the system’ and leveraging ‘vicarious power’ (Austin et al. 2006), which undermines transparency and standardisation. Such dependency on personal networks rather than institutional mandate reflects a system where influence is relational rather than procedural, creating vulnerability to bias and inconsistency.

Moreover, framing LTVNs as gatekeepers without granting them decision‐making autonomy exposes a structural incoherence. Authority and autonomy are not optional components of governance; they are foundational to accountability. The absence of a national tissue viability profile compounds this incoherence, enabling localised discretion that risks inequitable practice and fragmented standards. Patient safety and evidence‐based care are compromised by governance structures that impose responsibility without authority. This imbalance entrenches hierarchy, undermines accountability, and prioritises organisational politics over clinical integrity, making the absence of reform an active threat to care quality.

Product selection within healthcare is not merely a technical decision; it is embedded in organisational power structures that shape outcomes. Resistance from influential clinicians—often attributed to a limited understanding of the LTVN role—should not be viewed as incidental but as symptomatic of entrenched hierarchies that privilege certain professional identities. The reported bullying and intimidation (Wild et al. 2015), while ethically indefensible signals a deeper organisational failure: the inability to reconcile role expectations with authority structures. Managerial reluctance to support LTVNs when conflicts arise further perpetuates this imbalance, raising questions about accountability and governance.

Nurses, like other groups throughout history, have been described as an oppressed group having mostly female members (Peltomaa et al. 2013). Oppression theory suggests that powerlessness, lack of control over the working environment, and subsequent low self‐esteem contribute to the development of horizontal violence within the nursing profession and, as in other professions, it encompasses individual, social, and organisational characteristics (Wilson et al. 2011). Horizontal violence embodies an understanding of how oppressed groups direct their frustrations and dissatisfactions towards each other as a response to a system that excludes them from power (Hutchinson et al. 2006); within nursing, it is contended that nurses are dominated (and by implication, oppressed) by a patriarchal system headed by administrators, managers, and doctors. Nurses lower down the hierarchy of power may resort to aggression among themselves (Peltomaa et al. 2013). Nurses have been described as adopting the adaptive strategies of oppressed groups, directing their dissatisfaction inward towards each other, towards themselves and towards those less powerful than themselves, often the patients (Palviainen et al. 2003). Nurses control time for medicine, for food, for washing, for getting out of bed and back to bed, what dressing to use and how often it is done and when patients express an opinion or chose not to adhere to these shows of authority, they are labelled as non‐concordant (Morgan and Moffatt 2008).

TVNs are constrained in their ability to be autonomous and are not supported in a difficult role; they gate keep in order to prevent uncomfortable conversations and in doing so, they exert authority and power over less experienced staff in the organisation. It has been said that in doing so, they disempower them (Austin et al. 2006) with the ultimate consequence of disengaging staff from the opportunity and responsibility of contributing to the wound decision‐making process; thus, there is a real danger that oppression is perpetuated. LTVNs are caught up in this circle of power and manipulation in order to keep control of the standards and quality of wound care undertaken in their organisation.

Autonomy is often portrayed as a defining characteristic of nursing and a powerful professional ideology (Traynor 2019). It implies authority to make decisions and freedom to act based on professional judgement, which is grounded in both experiential and formal knowledge. However, this idealised notion of autonomy, emphasising authority, independence, and clear occupational boundaries, rarely aligns with the lived reality of LTVNs. Despite aspirations towards autonomy, their practice is constrained by managerial oversight, bureaucratic systems, efficiency targets, and the influence of other dominant professional groups. Rather than autonomy being inherent to their advanced role, LTVNs must actively negotiate and cultivate relationships to secure organisational authority. This raises critical questions about whether autonomy in nursing is genuinely attainable or merely rhetorical. The absence of medical supervision, traditionally a source of power, may paradoxically weaken their position, forcing reliance on inter‐professional collaboration to fulfil role requirements. Furthermore, while knowledge is often equated with power, the specialised expertise of LTVNs remains marginally recognised, with organisational structures lagging behind clinical advancements. This disconnect suggests that autonomy in this context is less a reality and more an aspirational construct, undermined by systemic and cultural barriers.

The difficulties of producing evidence for wound care have exacerbated the requirement for LTVN's to gate keep which product makes it onto to their WMFs. LTVNs expertise is grown from experience and education rather than research and decisions are made in the face of uncertainty where there is missing, unreliable, conflicting, or complex information (Madi et al. 2019). The lack of empirical evidence limits the ability of LTVNs to always articulate a rationale that is acceptable to the traditional and strict NHS patriarchal hierarchy. Their expertise is based on intuition, their ability for quick thinking (seeing the past and the future); seeing what is invisible to the generalist nurse and the ability to analyse rationally challenging situation (Melin‐Johansson et al. 2017). LTVN posts are lead nursing posts. They have to ‘navigate expectations’, referring to having difficult conversations with colleagues when they believe that quality of care delivery is impacted, and/or standards of care have been breached as in the case of the issues surrounding dressing procurement. They also have to ‘maintain order’, referring to actions that are used to prevent, contain, and manage colleagues' potentially negative behaviours as in the case of monitoring dressing usage to ensure concordance with their organisation's WMF.

Currently, the NHS lacks a standardised core job description for Tissue Viability Nurses or LTVNs, which raises significant concerns about role clarity and professional development. Ousey et al. (2014) highlight systemic inconsistencies across the UK in employment conditions, remuneration, role expectations, and training frameworks for these positions. This fragmentation extends to entry requirements for the specialty and the competencies and educational standards expected, creating ambiguity and potential inequity in career progression. While it seems logical to conceptualise junior TVN roles as developmental posts, where clinical expertise and academic knowledge are progressively acquired before promotion, the reality diverges sharply. Structural rigidity within NHS pay banding and hierarchical funding models, as noted by Wightman (2020), constrains such flexibility, thereby impeding the creation of transitional roles that could support workforce sustainability and skill acquisition.

Our findings reveal that LTVNs function within a highly interdependent and hierarchical care system, where they must continuously negotiate and assert their clinical expertise, professional authority, and autonomy to maintain control over wound management processes. This negotiation occurs across multiple levels: individual (direct patient care), decisional (interactions with senior and influential colleagues), and organisational (implementation of WMF). While LTVNs demonstrate a strong internalised sense of professional competence, this expertise is often under‐recognised by others, creating a persistent need for strategic positioning. Consequently, LTVNs employ mechanisms such as cultivating professional alliances and leveraging organisational tools, particularly for the WMF, to influence decision‐making and safeguard optimal wound healing outcomes. Their role demands the construction of a distinct professional identity situated at the intersection of competing professional logics and organisational imperatives within a complex system of care delivery.

### 6.1 Limitations

In this study, Scotland, Northern Ireland, or Wales were not represented as they have a different health care system, for instance they have national WMF with centralised systems for obtaining dressings and so LTVNs here are not required to gatekeep. This study recruited LTVNs from England, as there are, excluding ambulance Trusts, over 215 Trusts delivering care in the UK (The Kings Fund 2022). It is unclear how many Trusts have a Tissue Viability team, but it is assumed that most Trusts will have a TV service. Therefore, only a small proportion of LTVNs were interviewed.

### 6.2 Recommendations for Further Research

The agency of Lead Tissue Viability Nurses enabling them to perform their role effectively should be investigated further using a larger sample. Additionally, it is possible that lead Tissue Viability Nurses working in smaller district hospitals might have a different experience in their ability to influence how their service is developed and this merits further investigation.

## 7 Conclusion

Our study highlighted the lack of agency, authority and autonomy that impacts on LTVNs' ability to meet what is demanded of the role. Their knowledge and expertise if often underutilised. In order to maintain some power within their organisation and on national programmes that affect their working lives, they have to forge links with more powerful staff, fixing problems for those in power that could have been avoided if their voices had been listened to and engaging in gate keeping behaviours that unwillingly perpetuate hierarchical and patriarchal oppression in nursing. We join Austin et al. (2006) in highlighting that if LTVNs are to reach their full potential and use their expertise to inform the practice of others, organisations need to recognise these posts as central to practice development. In particular a framework is required which enables LTVNs to use more inclusive approaches when seeking to bring about changes in practice that allow them to be confident that they are supported in their endeavours. Our paper adds to the literature base by highlighting the multiple facets of the role and the complex environments impacting upon it and call for the development of a professional and organisational identity that will offer the autonomy, authority, and freedom to act that this senior nursing role commands.

## Author Contributions

Made substantial contributions to conception and design, or acquisition of data, or analysis and interpretation of data: F.P., M.L., T.R. Involved in drafting the manuscript or revising it critically for important intellectual content: F.P., M.L., T.R. Given final approval of the version to be published. Each author should have participated sufficiently in the work to take public responsibility for appropriate portions of the content: F.P., M.L., T.R. Agreed to be accountable for all aspects of the work in ensuring that questions related to the accuracy or integrity of any part of the work are appropriately investigated and resolved: F.P., M.L., T.R.

## Funding

The authors have nothing to report.

## Ethics Statement

The University of Northumbria granted ethical approval (Ref. 22867).

## Conflicts of Interest

The authors declare no conflicts of interest.

## Data Availability

The data that support the findings of this study are available on request from the corresponding author. The data are not publicly available due to privacy or ethical restrictions.

## Appendix 1

**Table:**

No.	Topic	Item	Tick
*Title and abstract*			
S1	Title	Concise description of the nature and topic of the study. Identifying the study as qualitative or indicating the approach or data collection methods is recommended	☑
S2	Abstract	Summary of key elements of the study using the abstract format of the intended publication. Typically includes background, purpose, methods, results and conclusions	☑
*Introduction*			
S3	Problem formulation	Description and significance of the problem/phenomenon studied; review of relevant theory and empirical work; problem statement	☑
S4	Purpose or research question	Purpose of the study and specific objectives or questions	☑
*Methods*			
S5	Qualitative approach and research paradigm	Qualitative approach and guiding theory if appropriate identifying the research paradigm is also recommended; rationale	☑
S6	Researcher characteristics and reflexivity	Researchers' characteristics that may influence the research; potential or actual interaction between researchers' characteristics and the research questions, approach, methods, results and/or transferability	☑
S7	Context	Setting/site and salient contextual factors; rationale	☑
S8	Sampling strategy	How and why research participants, documents or events were selected, criteria for deciding when no further sampling was necessary; rationale	
S9	Ethical issues pertaining to human subjects	Documentation or approval by an appropriate ethics review board and participant consent, or explanation for lack thereof; other confidentiality and data security issues	☑
S10	Data collection methods	Types of data collected; details of data collection procedures; rationale	☑
S11	Data collection instruments and technologies	Description of instruments and devices used for data collection; if/how the instrument(s) changed over the course of the study	☑
S12	Units of study	Number and relevant characteristics of participants, documents or events included in the study; level of participation (could be reported in results)	☑
S13	Data processing	Methods for processing data prior to and during analysis	☑
S14	Data analysis	Process by which inferences, themes, etc. were identified and developed; usually references a specific paradigm or approach; rationale	☑
S15	Techniques to enhance trustworthiness	Techniques to enhance trustworthiness and credibility of data analysis; rationale	☑
*Results/findings*			
S16	Synthesis and interpretation	Main findings; might include development of a theory or model or integration with prior research or theory	☑
S17	Links to empirical data	Evidence to substantiate analytic findings	☑
*Discussion*			
S18	Integration with prior work, implications, transferability, and contribution(s) to the field	Short summary of main findings; explanation of how findings and conclusions connect to, support, elaborate on, or challenge conclusions of earlier scholarship; discussion of scope of application/generalisability; identification of unique contribution(s) to scholarship in a discipline or field	☑
S19	Limitations	Trustworthiness and limitations of findings	☑
*Other*			
S20	Conflicts of interest	Potential sources of influence or perceived influence on study conduct and conclusions, how these were managed	☑
S21	Funding	Sources of funding and other support; role of funders in data collection, interpretation and reporting	☑

*Source:* Adapted from O'Brien et al. (2014).
